# Urban Health and Nutrition Day Or Only Immunisation Day? Barriers and Bottlenecks in Implementing Urban Health and Nutrition Day in An Urban Primary Health Centre of Nagpur, Central India

**DOI:** 10.34763/jmotherandchild.20212501.d-21-00006

**Published:** 2021-10-11

**Authors:** Sitikantha Banerjee, Kalaiselvi Selvaraj, Kajari Bandyopadhyay, Mubashshera Firdous Khan, Tikesh Bisen, Pradeep Deshmukh

**Affiliations:** 1 Department of Community Medicine, AIIMS Nagpur, Nagpur, Maharashtra, India; 2 Model UPHC Project, Tata Trusts, Nagpur, Maharashtra, India

**Keywords:** Slum, Urban Health Nutrition Day, barrier immunisation, programme evaluation

## Abstract

**Background:**

This study was carried out to evaluate the implementation status of Urban Health and Nutrition Day (UHND) and to explore barriers and bottlenecks as perceived by community-level service providers in the selected city of Nagpur, Maharashtra.

**Material and methods:**

This mixed-method study was conducted using a triangulation design. An initial gap assessment was carried out quantitatively using an observation checklist. Focus group discussion among ASHAs and interviews among frontline health workers involved in community mobilisation were carried out to understand the bottlenecks and barriers.

**Results:**

Supplies of drugs and logistics, like paediatric iron and folic acid tablets, calcium tablets, and weighing machines, were inadequate. Services like distribution of conventional contraceptives, testing for malaria, urine albumin/sugar, haemoglobin estimation, screening for diabetes and hypertension, sputum collection, and qualitative iodine test kits are not available in any of the UHND sessions. Major challenges in the implementation of UHND were found to be as follows: unserved areas and left-out urban slum pockets; the distribution paradox of UHND location with an ill-defined geographic boundary; restriction of range of services to antenatal registration and immunisation with gross neglect of other components; suboptimal training of staff; insufficient availability of space, logistics, and health manpower; non-involvement of community members and Urban Local Bodies; and poor monitoring and supervision.

**Conclusion:**

The conduct, use, and list of services offered in UHND are far from the desired goal. For optimal use, reallocation of the UHND sessions, preferably in unserved and underserved slums, need to be carried out after suitable vulnerability assessment. Integration of the Health, Women, and Child Department and Urban Local Bodies is required for convergent planning, monitoring, and supervision.

## Introduction

Over the last decade, India has experienced rapid and unplanned urbanisation. Due to the migration of poor people from rural to urban areas to maintain a livelihood, most cities in India have seen a constant rise in slum populations [1,2]. Increasing population disproportional to the available resources, preexisting occupational and social vulnerability and poorly developed health infrastructure have led to poor health outcomes among slum populations in Nagpur, Maharashtra [3,4]. The majority of the health indicators, including childhood illnesses, malnutrition and infant and under-five mortality rate, are worse among the urban poor (i.e., mostly slum-dwellers) when compared to their non-slum-dwelling counterparts [5]. Despite better availability of health facilities in urban regions, inequity in distribution resulting in lack of health access for slum-dwellers leads to poor health status among them.

The Government of India launched National Urban Health Mission (NUHM) in 2013 to streamline health care delivery systems in urban areas, with an emphasis on slums [6].

Under NUHM, urban primary health centres (UPHCs) are the first point-of-contact fixed facilities to provide comprehensive health services on an outpatient basis. However, since it was felt that fixed facilities could not meet the health needs of all urban people, especially in densely populated slums, Urban Health and Nutrition Day (UHND) was introduced by NUHM as a routine outreach activity to provide a convergence platform where a package of preventive, promotive and basic curative services could be provided by community health workers (CHW) at the doorsteps of the urban poor. UHNDs also provide an opportunity to improve community empowerment and ownership through an Accredited Social Health Activist (ASHA) and Mahila Aarogya Samiti (MAS) members from the community [7,8]. Thus, the smooth functioning of UHND is of utmost importance to improving the overall health of the urban poor [9].

NUHM activities are being implemented in phases in various cities of India. In Nagpur, it is in a nascent stage, operationalised since 2016. Anecdotal evidence from monthly reports of auxiliary nurse midwives (ANM) reflects that implementation of UHNDs is not as per recommendations. To date, to the best of our knowledge, no implementation research or external monitoring has been conducted in the current setting to assess the actual performances of UHND. Further, the perspectives of CHWs regarding bottlenecks in implementing the UHND have not been explored. Any endeavour to streamline the conduct of UHND requires baseline information. With this background, this study was conducted with the aim of evaluating the current implementation status of UHND sessions in areas served by selected UPHCs in the Nagpur district of Maharashtra and to explore perceived bottlenecks and solutions from the CHWs’ point of view.

## Material and methods

### Study setting

The study was conducted at Nandanvan UPHC, located in Nagpur, which caters to about 80,000 people, including 52,000 slum dwellers. The community-based outreach services of Nandanvan UPHC are provided through 28 UHNDs, which are held once a month on pre-specified dates. They are usually organised in Anganwadi centres (AWCs), primary schools or other suitable community spaces, where a package of services are provided by Anganwadi workers (AWW) and ANM, whereas community mobilisation is carried out by ASHA and MAS members. One nongovernmental organisation (NGO) also works in collaboration with health authorities to coordinate UHND activities. To get a broad insight into the study topic, professional diversity was considered in selecting the participants. ANMs, ASHAs, AWWs and an NGO representative involved in the UHND sessions were included as participants in the qualitative inquiry and are termed as CHWs.

**Study design, tools and data collection techniques**

This mixed-method study was conducted using a triangulation design, and data were collected between November 2019 and January 2020.

**Sampling methods**: In the selected UPHC, a total of 28 UHND sessions were planned in a month. Without any sampling, all these UHND sessions were visited for participant observation as per the schedule submitted in UPHC. Each UHND session was observed by a trained public health expert from initiation to the end of the session.

For the qualitative interviews, all ANMs, staff nurses involved in service delivery, were included, and ASHAs were approached based on their expressed willingness to participate in the study. An initial gap assessment was carried out quantitatively using an observation checklist as the first study tool to assess quality of care. The checklist was prepared according to the guidelines proposed by NUHM for conducting UHND [9]. Data were filled by trained epidemiologists for each observed session. Terms of references (TOR) included domains under the implementation of maternal and child health care; curative care for common conditions; screening and continuity of care for NCDs; raising awareness toward preventive and promotive health; provision of family planning services; and access to essential drugs and testing facility. To assess recent regularity and use of services, data on regularity of sessions, number of beneficiaries in each session, due list and follow-up details, availability of logistics and so on were obtained using a structured data extraction form from the last six months of reports prepared by ANMs.

After quantitative assessment, qualitative techniques were used to understand the bottlenecks and barriers in implementing the services. Researchers trained in qualitative methods collected the data. In-depth interviews (IDIs) were carried out among eight purposively selected ASHAs, eight AWWs, four ANMs and one NGO representative, all in the regional language, using a pre-tested, unstructured interview guide as second study tool. An IDI guide included questions related to all the terms of reference. Interviews were continued until data saturation. Field observations and IDIs were followed by focus group discussion (FGDs) among ASHAs. After the briefing on the objectives of the exercise, two free-listing exercises were conducted involving ASHAs on: (1) Activities carried out in their UHND session, and (2) activities expected to be carried out in UHND. After free listing, one FGD was carried out among 11 ASHAs present on that day with the help of a predesigned FGD guide (the third study tool). The domains unearthed in the IDIs were used in the FGD guide. Each TOR was discussed among the group, and for each domain, gaps were identified, possible reasons and ways to address such gaps as perceived by ASHAs were considered. Multiple probes were given in the vernacular language whenever clarification was needed. The entire FGD exercise lasted 90 minutes and was audio recorded; a transcript was prepared on the same day by the person who took notes in the FGD.

**Figure 1:**
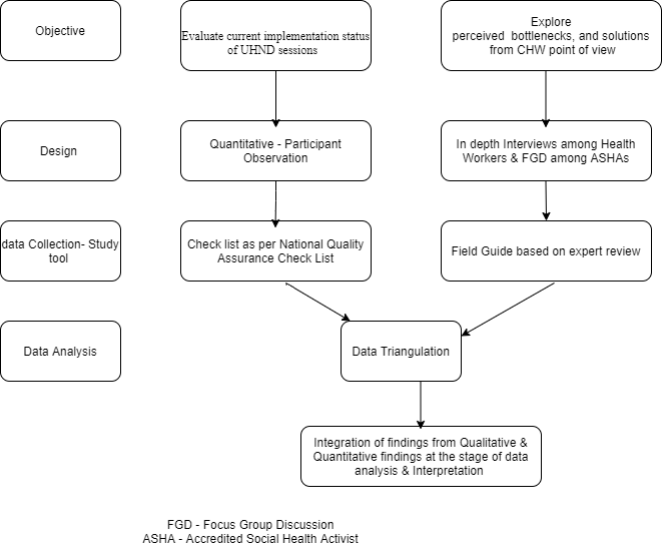
Flow of integration of qualitative and quantitative methods in data collection and analysis

The flow of integration of qualitative and quantitative methods in data collection and analysis is depicted in Fig 1.

### Data analysis

The proportion of UHND sessions providing each component of services was presented by number and percentage. The free listing exercise on expected and actual services delivered in UHND was analysed in Anthropac to get the salience score. Smith’s salience score is an index that indicates the importance and centrality of the specific item. A score close to 1 indicates high salience, and a score close to zero indicates less salience [10]. The audio-recorded clips were transcribed into English and read several times iteratively by two independent researchers. Identification of relevant codes and thematic analysis content was carried out manually to identify patterns of meaning across the transcripts.

### Ethics

Written informed consent was obtained from each participant before each interview. To ensure privacy, the confidentiality of the opinion and participants’ comfort, interviews were conducted at a place and time convenient to them, and no identifiers were included in the transcript. The transcripts and audio-recording clips were stored in password-protected files accessible only to the study investigators. The study was undertaken after obtaining clearance from the Institute Ethics Committee of All India Institute of Medical Sciences Nagpur (Reg No-EC/Dept of Comm Med/AIIMS/Nagpur, Dated 21/02/19).

## Results

A total of 28 UHND sessions were observed and findings from this evaluation are organised as per the following:

### UHND implementation status

**Regularity and venue of the UHND sessions**: Of the 28 sessions, two were not conducted on the scheduled date, as the ANM was on leave. Schedules of four more sessions needed to be changed due to the nonavailability of staff over the last six months. Missed sessions and changes of venue over six months were found in one and three sessions, respectively. Of the 28 sessions, 22 were organised at AWCs; five were in a private building; and one was in a community garden maintained by the municipality. No dedicated toilet or water supply was available at session sites. In none of the sessions were MAS members or Urban Local Body (ULB) representatives present.

**Display of Charter and IEC materials**: Fixed display boards were found in only one session, though that had only immunisation-related information. In other sessions, ANMs carried one poster of Mission Indradhanush [11], which described immunisation services only. Other services available (other than immunisations) were not displayed in any session. Health education materials were displayed in all the 22 sessions organised in AWCs, but not in any session organised in other settings.

**Logistics issues**: No contraceptives were found to be distributed to the beneficiaries in any session. At none of the session sites were essential items for examinations available, such as examination tables, screens for privacy, stethoscopes, inch tape, MUAC tape, and diagnostic kits. Details of drugs and logistics available at UHND are depicted in Tables 1 and 2, respectively. The adult weighing machine was available in 22 sessions, and 19 were functioning. Further, at 16 sites (57.1%), HCWs were using it to measure the weight of pregnant women. Salter’s scale or paediatric weighing machine was available in 22 sessions, and for 17 sessions they were in usable condition; nine (32.1%) were used for measuring the children’s weight. In two sessions adult weighing machines were used to measure the baby’s weight. Similarly, height scales were available in 20 sessions; however, in only five (17.9%) sessions were height measurement actually carried out.

**Table 1:** Availability of drug and contraceptives at UHND sessions

Sl No	Name of the drug that need to be supplied	Current availability status	Comment
1	ORS sachet	Available	Regular supply^#^
2	IFA tablet	Available	Regular supply*
3	IFA tablet (small)	Not available	Not supplied
4	IFA syrup	Not available	Irregularly supplied
5	Anti-malarial syrup/tablet	Not available	Not supplied
6	Cotrimoxazole tablet/ syrup	Not available	Not supplied
7	Paracetamol tablet	Available	
8	Pediatric Paracetamol syrup	Not available	Not supplied
9	Albendazole tablet/ syrup	Available	Regular supply
10	Zinc tablet	Not available	Supplied, but not brought to the session by ANM; not available in the field.
11	Calcium tablet for pregnant women	Not available	Irregular supply
Contraceptives:			
1	Mala-N	Not available	Not supplied
2	Chhaya (Centchromen)	Not available	Supplied to UHTC, but asked by Municipality to return back to store; no supply in this moment
3	Antara (Medroxyprogesterone injection)	Not available	Injection given at UHTC; not supplied for field use
4	Condom	Not available	Supply available at UHTC; not distributed to ASHA/ANM till date.
5	Emergency Contraceptive Pills	Not available	Available at UHTC; not supplied for UHND session

* Not for distribution among lactating mothers. #Supply in last six months was considered for assessing regularity.

**Table 2:** Distribution of UHND sessions according to the availability of logistics in the session: (n=28)

Sl No	Logistics that need to be available	Number of sites where available (%)	Comment
1	Hand gloves	28 (100)	Limited number of gloves available
2	Red bag for disposal	28 (100)	
3	Black bag for disposal	28 (100)	
4	Absorbent cotton	28 (100)	
5	Cotton bandage	0	
6	Blank MCP cards	28 (100)	
7	Due list of beneficiaries	28 (100)	
8 9	Adult weighing machine available Adult weighing machine functioning properly and calibrated	22 (78.6) 19 (67.9)	All the six sessions organised outside AWCs surement don’t facilities have weight/height mea-
10	Salter’s scale/digital paediatric weighing machine	22 (78.6)	
11	Salter’s scale/digital paediatric weighing machine in usable condition available	17 (60.7)	In five sites machine either out of order or not calibrated
12	Height measurement scale (any type)	20 (71.4)	
13	Examination table, screen for privacy, stethoscope, digital sphygmomanometer, , foetoscope, thermometer, inch tape, MUAC tape, haemoglobin testing kit, RDK kit, needle/lancet, pregnancy testing (Nischay) kit, urine testing kit (Uristix), test tube, urine collection container, sputum collection container, IMNCI chart booklet	0	Digital sphygmomanometer pregnancy testing (Nischay) kit: available in UPHC, but not brought in UHND session site

### Use of UHND and range of services offered

**Figure 2:**
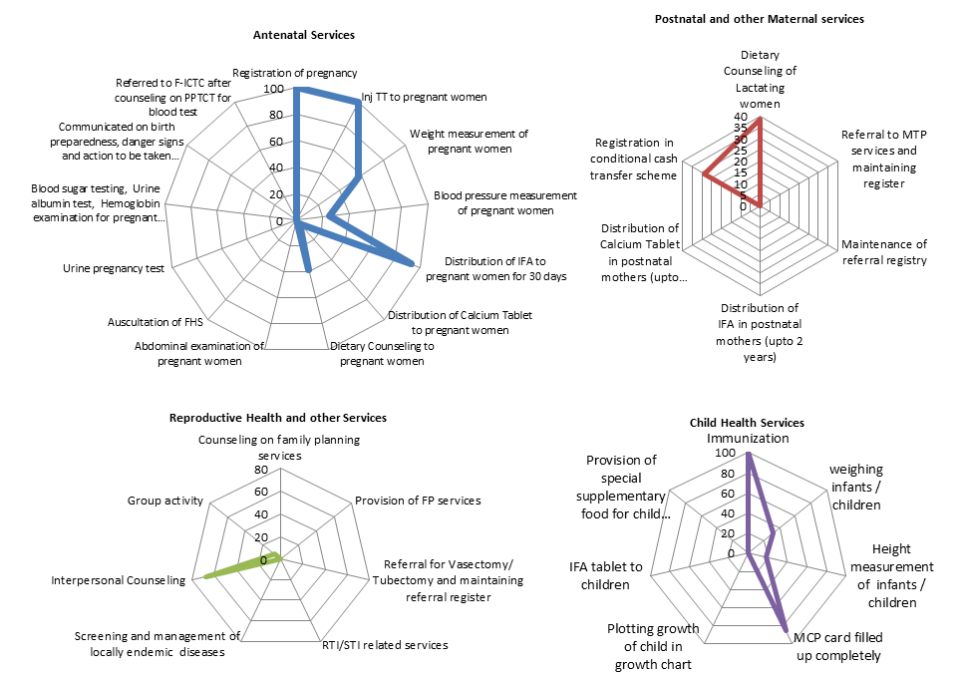
Services provided in UHND session

Number of beneficiaries attending UHND varied based on location (slum vs. non-slum). Median (IQR) attendance in non-slum UHNDs was seven (4–15) as against 52 (18–65) in slum UHNDs. All sites offered services like registration of pregnant women, immunisation of pregnant women and children under five years of age, and distribution of iron folic acid (IFA) and calcium tablets to pregnant women. Details of key services provided at UHND sessions, as described in Fig. 2 revealed that antenatal services are predominately restricted to pregnancy registration and IFA tablet distribution, whereas among postnatal services only dietary counselling and registration of conditional cash transfer schemes were carried out. Among child health service components, only immunisation and filling of MCP cards were carried out. No reproductive health services were available except interpersonal counselling. Basic investigations such as testing for malaria, urine albumin/ sugar, haemoglobin estimation, screening for diabetes and hypertension, sputum collection and qualitative iodine test kits were not available in any of the UHND sessions. As depicted in the result of the free listing, most of the CHWs opined that the outreach sessions are organised to provide immunisation and registration of pregnant women (salience score of 0.786 and 0.510, respectively). Other components of UHND were grossly ignored by the providers. (Table 3.)

**Table 3:** Activities actually performed and that need to be made available at UHND session: free listing as per ASHA’s perspective

Activities performed in a routine UHND session				Activities that should be performed/facilities that need to be available			
Sl No	Items	Frequency (%)	Salience	Sl No	Items	Frequency (%)	Salience
1	Immunisation for 0-5 yr child	91.7	0.786	1	Supplementary nutrition for children	33.3	0.319
2	Registration of pregnant mother and providing MCP card	75.0	0.510	2	Involving beneficiaries not having permanent address	33.3	0.222
3	Educating mother on newborn and child care, nutrition	83.3	0.351	3	BP measurement	33.3	0.161
4	Antenatal care	33.3	0.300	4	Ensure supply of floor mat	16.7	0.150
5	Inj TT to pregnant women	41.7	0.296	5	Calcium supplementation	25.0	0.144
6	TT injection for 10 and 15 yr child	33.3	0.142	6	Proper sitting arrangement and space	33.3	0.143
7	Educating antenatal women on diet and other issues	33.3	0.135	7	IFA supplementation	25.0	0.141
8	IFA distribution to pregnant women	16.7	0.127	8	Ensure supply of weighing machine	16.7	0.117
9	Advising postnatal women on contraceptives	25.0	0.103	9	Health education/counselling session	16.7	0.117
10	Information on government schemes	16.7	0.093	10	Supplementary nutrition for pregnant mothers	16.7	0.111
11	Searching and vaccinating left-out population	16.7	0.090	11	Ensuring drinking water facility	16.7	0.108
12	Reminder for immunisation	16.7	0.080	12	Weighing of children	16.7	0.107
13	Weighing of pregnant women	16.7	0.074	13	Health camp	8.3	0.083
14	Registration for family planning	8.3	0.073	14	Separate room for check-up of pregnant mothers	8.3	0.083
15	Postnatal care	8.3	0.063	15	Medical check-up by doctor	8.3	0.083
16	Calcium distribution to pregnant women	8.3	0.046	16	Nutritional counselling	8.3	0.083
17	Weighing of children	16.7	0.026	17	Weighing of pregnant women	16.7	0.081
18	Ask ANC mother to bring Adhar card	8.3	0.014	18	paediatric facility	8.3	0.067
19	Giving vitamin A	8.3	0.014	19	Ensure supply of height measurement scale	8.3	0.050
20	Advise on hygiene	8.3	0.012	20	Iron supplementation for children	8.3	0.042
				21	Deworming	8.3	0.028
				22	Distribution of syrup for children	8.3	0.019
				23	Diagnostic/testing facility	8.3	0.017
				24	Temperature check	8.3	0.014
				25	Antenatal check up	8.3	0.009

### Bottlenecks and barriers in implementing UHND (qualitative exploration)

Thematic analysis of the transcripts revealed the following bottlenecks and barriers faced by the CHWs.

### Distribution paradox of UHND location

Striking variation in the number of beneficiaries per UHND session has been noted in the slum and non-slum settings. In all five sessions organised in non-slum-predominant areas, poor community involvement and compliance have been noted. One of ANM said, “Most of the people here prefer private practitioners. Even those who go to the government facility, they get all the facilities in the UPHC which is five minutes from here.” The ASHAs seconded this and added, “The number of beneficiaries who are not attending the sessions remain very high during every month. To cover this up, I need to make home visits for counselling and tracking their immunisation status. That increases my workload manifold.”

### Ill-defined geographic boundary with unserved or over-served areas

The geographic boundary of each UHND location was not clear-cut to the service providers. When data collection was done for this study, they did not have a line list of beneficiaries. For ease of work, CHWs divided the population catered to by the said UPHC arbitrarily into some “areas” catering to 1,000 to 1,200 people, roughly based on the number and residence of ASHAs. However, due to constant in-migration, such areas have been expanding, posing great challenges to existing manpower. Often, the CHWs were not being updated regarding slums included or excluded. This resulted in some unserved and left-out pockets as well as some duplication of beneficiaries. When ASHAs were asked about their beneficiary lists, it was observed that in four areas the number of beneficiaries was much less (<50%) than expected (i.e., as calculated based on area population and age distribution) [12]. ANMs also reported that many people come to UHND sessions from adjacent slum areas, outside the catchment area, where no UHNDs are carried out. The ANMs/AWWs had to provide services to these attendees without entering it in the record or portal. Thus a true picture is not being reflected in the records.

### Range of services

As depicted in the free listing (Table 3), most of the CHWs opined that the outreach sessions were organised to provide immunisation and registration of pregnant women (salience score of 0.786 and 0.510, respectively). Other components of UHND were grossly ignored.

### Training and knowledge of staff

Though the basic Module of ASHA [13] was expected to cover the scope for UHND, some ASHAs said, “We did not undergo any formal training on different components of UHND.” Many of them are not aware of all the components of UHND. In free listing their perceptions on the services that need to be available, very few participants listed components like supplementation of iron syrup to children or deworming (salience score of 0.042 and 0.028, respectively). Also, few ASHAs reported that “they felt UHND as a separate program organised on a different date in AWCs.”

### Session plan

A tendency to complete all the sessions before the 15th of every month was observed. On exploration, they replied, “We plan our sessions to complete within the initial two weeks so that we can utilise the remaining days of the month for preparing the reports. Further, if I can finish up my quota, I can take charge of that of one of my colleagues, who might be on leave or deputed for some other work, which happens very commonly.” Except for two sites where the supervisor from the central immunisation team had visited for quality assurance, none of the other sites had regular supervision and monitoring. A medical officer of UPHC commented, “Since some of the days in the first two weeks do have even three sessions on the same day, monitoring or logistic adjustment becomes challenging.”

### Space crunch

With the majority being located inside slums, the UHND session sites are plagued with a space crunch. Nine out of the 28 sessions could barely accommodate five or six adults and those sessions conducted in open places had privacy issues. One ANM said, “We cannot carry out any abdominal examination for the pregnant ladies. We conduct sessions even in an open park/courtyard, which does not have privacy or logistics. How will it be possible then? Moreover, during monsoon, it becomes almost impossible to carry out the sessions.”

### Availability of logistics

The supply of supplements (like IFA, calcium and vitamin A) were found to be irregular and inadequate. “The amount of calcium tablets supplied gets over within one to two sessions and most of the beneficiaries don’t get it. … [S]upplies of iron tablets are not adequate. If beneficiaries are already taking iron tablets from private, we have to ask them to continue so,“ commented one ANM. Due to insufficient supply, sometimes CHWs distributed fewer tablets than should be according to norms. Home delivery of iron and calcium tablets by ASHAs to pregnant and lactating women were not carried out.

### Involvement of MAS and Urban Local Bodies (ULBs)

No MAS member was found to be present in any UHND session. On exploration, the coordinator for the MAS replied, “Development of MAS is in a very primitive stage in most of the slum areas. The liaison between the ASHAs and the MAS members are not in place and we are in process of addressing this issue, sometimes by redistributing the MAS members.” ASHAs and ANMs have no idea about the role of MAS in UHND. Representatives from Urban Local Bodies (ULB) did not interact with CHW in coordinating UHND activities. Lack of community awareness on services provided in UHND and very poor community engagement were observed.

### Other issues

Frequently, ASHAs and ANMs were involved in other survey/ services, which resulted in discontinuation of routine services. Numbers of ASHAs present were much fewer than required per the NUHM norm. Lack of coordination between ASHA, AWW and ANM and internal conflict about job distribution among them was also noted. Currently, contraceptives are expected to be distributed and monitored through Family Planning–Logistics Management Information System (FP-LMISF).^14^ Training on this online portal was given to only select CHWs. According to one ANM, “ASHAs are unable to use this system as they did not have training and they do not understand its functionality. All don’t have a smartphone to use this application. The IT support team did not provide user-ID for everyone.”

## Discussion

In this study, the current status of the UHNDs’ functioning was evaluated along with the barriers CHWs perceived to implementing services. The supply of logistics and space was inadequate to deliver the package of services. Moreover, lack of clarity on the part of CHWs about their role, technical inefficiency, internal conflict on job distribution and coordination, poor community participation and community ownership were found to be major bottlenecks. As a result, only a few components (immunisation and pregnancy registration) are provided. There are few published studies in India that evaluate UHNDs. Though operational challenges are largely context-specific and noncomparable between different settings, some common issues have been raised during the evaluation of UHND in other settings. Tripathy et al. evaluated UHND sessions in slums in Berhampur, Odisha, and observed poor AWW involvement, inadequate space and logistics and poor supervision hindering its optimum performance [15]. In a process evaluation survey in Vadodara, Gujarat, Mehta et al. observed that identification of high-risk pregnancy, abdominal examination, blood pressure measurement and haemoglobin estimation were not carried out in UHND.^16^ Kotecha et al. evaluated UHND sessions at Bhavnagar, Gujarat, and found inadequate tracking of drop outs, failure to update growth charts and unsatisfactory performance by ANMs delivering services [17]. Supply of vaccines was adequate (100%) in this study setting, but it was found to be 70% and 94.7% in studies carried out at Uttarakhand and Vadodara, respectively [16,18].

The main strength of this study lies in its qualitative design for an in-depth understanding of the health care provider’s perceptions of barriers. The inclusion of varied occupational groups helped collect deeper insights into the topic. The results should be interpreted with limited external validity, as UHNDs of only one UPHC were studied here, and most of the challenges might be context specific. As providers’ behaviour may change in the presence of an observer, the possibility of a Hawthorne efect cannot be ruled out. Further, including more UPHCs would have given a broader perspective.

Based on the study findings, the following recommendations can be made: Reallocation of the UHND sessions need to be carried out, preferably in unserved and underserved slums, after suitable vulnerability assessment. There is also an imperative need for scaling up the number of AWWs to avoid the sealing effect. In liaison with ULBs, appropriate places have to be identified, with appropriate provision of logistics and privacy measures. Wherever alternate arrangements cannot be made, ANC women have to be referred to the nearest UPHC. They need to be followed up at their residence by ASHA. Planning and demarcation of areas for individual ASHAs needs to be done. The routine annual enumeration exercise of ASHAs needs to be strictly monitored by AWWs and medical oficers to prevent leaving out slum areas or urban pockets. The citizen charter should be displayed at the UHND session site, clearly depicting the package of services offered in UHND session, date of the session, and contact number of ANM and ASHA for consultation. Supply of drugs (contraceptives, supplements, etc.) and logistics should be ensured for the proper functioning of UHND. Buying materials for sitting arrangements and other low-cost essential logistics can be mobilised from the unutilised fund for MAS members. To expand the services of UHND, all concerned stakeholders (ANMs, ASHAs, MAS members, and MOs) need to be oriented to its scopes and package of services. ASHA and MAS members should be suitably trained in screening for NCDs and basic lab investigations such as rapid diagnostic kits for malaria, haemoglobin estimation, iodination of salt and so on. Nearby designated microscopy centres have to be tied up for making arrangements for outreach sputum collection centres at UHND. The session schedule should be revised, which should spread over all the days instead of the initial two weeks. The duration of the sessions should be increased, and the medical oficer should conduct periodic visits to the sessions.

## Conclusion

To sum up, current UHND services focus primarily on registration of pregnancy, vaccination of children, and pregnant women, whereas other components are grossly neglected. Integration of Health, ICDS, and Urban Local Bodies is required for convergent planning, monitoring and supervision. The provision of a full range of services is urgently needed to improve the health status of this vulnerable slum population.

## Key points

1. The package of services under Urban Health and Nutrition Day (UHND) are partially implemented, focusing primarily on pregnancy registration and vaccination, whereas other essential components—like growth monitoring, contraceptive services—are grossly neglected.
2. Intersectoral coordination is grossly lacking, and there is poor coordination between community health workers and Urban Local Body and ICDS staff.
3. Distribution paradox of UHND sessions reflects an urgent need to reallocate the session sites, preferably in unserved and underserved slums, after suitable vulnerability assessments.
4. The routine annual enumeration exercise of ASHAs needs to be strictly monitored by AWWs and medical officers to prevent left-out slum areas/urban pockets.

## Disclaimers

None.

## Declarations

- Ethics approval (include appropriate approvals or waivers): Ethical clearence was obtained from institute Ethics Committee.
- Consent to participate (include appropriate statements): A written informed consent was obtained from each participants before including them in this study.
- Consent for publication (include appropriate statements): This study has not been sent anywhere for publication and was not published anywhere. Consent was obtained from all the participants before sending this article for publication in this esteemed journal.
- Availability of data and material (data transparency): All the data collected and used in this study for analysis were kept in a secure passward protected system, which if required can be shared with the editorial board.
- Code availability (software application or custom code): In this study Microsoft Excel and Visual Anthropac-freelist were used, both being free software available in public domain.
